# Evaluation of protection induced by immunisation of domestic pigs with deletion mutant African swine fever virus BeninΔMGF by different doses and routes

**DOI:** 10.1016/j.vaccine.2017.12.030

**Published:** 2018-01-29

**Authors:** Pedro J. Sánchez-Cordón, Tamara Jabbar, Margot Berrezaie, Dave Chapman, Ana Reis, Patricia Sastre, Paloma Rueda, Lynnette Goatley, Linda K. Dixon

**Affiliations:** a The Pirbright Institute, Ash Road, Pirbright, Woking, Surrey GU24 0NF, UK; b Inmunología y Genética Aplicada S.A. (INGENASA), Hermanos García Noblejas, 39, 28037 Madrid, Spain

**Keywords:** African swine fever, BeninΔMGF, Pigs, Immunisation routes, Immunoglobulins, Cytokines

## Abstract

A live attenuated African swine fever virus (ASFV) vaccine candidate, produced by deletion of several genes belonging to multi-gene families MGF360 and 505 from virulent Benin 97/1 strain (BeninΔMGF), induces protection in pigs against parental virulent strain. In order to better define the safety and efficacy of this attenuated vaccine candidate and to understand protective mechanisms, we extended previous studies by intramuscular immunisation of pigs with the deletion mutant BeninΔMFG at different doses (10^2^, 10^3^, 10^4^ TCID_50_), together with intranasal immunisation at the 10^3^ dose. Results demonstrated a strong correlation between both doses and routes of immunisation of BeninΔMFG and the percentage of protection achieved, the onset of clinical signs, the viremia levels reached and the onset of death in non-protected pigs. The results show that the intramuscular route using high doses (10^4^ TCID_50_) is the best option for immunisation. Only transient increase in temperature associated with a peak of virus genome levels was observed in most pigs after immunisation. Then, virus genome levels progressively decreased throughout the experiment until reaching low or undetectable levels in those protected pigs that survived after challenge. The IgM antibody responses following immunisation were detected between day 7–10 post-immunisation and remained at elevated levels for 10–18 days in most pigs before dropping. IgG was detected from day 15 to 21 post-immunisation and maintained at increased levels for the remainder of the experiment in most pigs. Induction of IFNγ and IL-10 was detected by ELISA in sera from some pigs immunised with 10^3^ TCID_50_ by intramuscular or intranasal route at early times post-immunisation. IL-10 was also detected in serum from some non-protected pigs included in these groups after challenge.

## Highlights

• Immunised pigs with BeninΔMGF were protected against parental virulent ASFV strain.
• To improve safety and efficacy new doses and routes of immunisation were tested.
• Intramuscular immunisation of high doses showed the best percentage of protection.
• A new ELISA detected specific IgM antibodies at early stages after ASFV infection.
• Early induction of IFNγ and IL-10 in vaccinated pigs probably critical to protection.

## 1 Introduction

African swine fever (ASF) is one of the most significant infectious diseases affecting the swine industry, with many isolates causing up to 100% lethality in domestic pigs. ASF is endemic in most sub-Saharan countries in Africa and in Sardinia. Since 2007 ASF has spread from Georgia in the Caucasus, to the Russian Federation and Eastern Europe including EU countries [1]. There is no vaccine for ASF and this limits disease control.

ASF is caused by a complex double-stranded DNA virus, African swine fever virus (ASFV), which encodes up to 167 genes [2], [3]. Many genes encode proteins with roles in evasion of host defence’s. Amongst these are proteins that inhibit type I interferon induction or responses including a TLR3 agonist, I329L, and members of MGF families 360 and 505/530 [4], [5], [6]. Levels of protection up to 100% against virulent virus challenge have been achieved by immunisation with attenuated ASFV. Deletion of multigene family members MGF 36-10L, 11L, 12L, 13L, 14L and 505/530 1R, 2R from the Pr4 isolate or MGF 360-12L, 13L, 14L and MGF 505/530 1R, 2R, 3R from the Georgia 2007 isolate [7] resulted in virus attenuation and induction of protection against challenge. We showed that deletion of these genes plus an additional deletion of MGF 505-3R and interruption of MGF 360-9L and MGF 505-4R from the Benin97/1 isolate (BeninΔMGF) also resulted in attenuation of the virulent Benin97/1 and induction of high levels of protection against virulent parental virus challenge [6].

In the current study we compared protection induced by intramuscular immunisation of pigs with the deletion mutant BeninΔMFG at different doses (10^2^, 10^3^, 10^4^ TCID_50_), together with intranasal immunisation at the 10^3^ dose. The aim was to better define the safety and efficacy of this attenuated vaccine candidate and to understand its protective mechanisms. Since the BeninΔMGF strain is genotype I, the major genotype circulating in West and Central Africa and Sardinia, this strain may be a potential vaccine strain in these regions and others if cross-protection against other genotypes is confirmed as demonstrated for the OURT88/3 attenuated genotype I strain [8].

## 2 Materials and methods

### 2.1 Viruses, animals and experimental design

The preparation of viruses used, Benin97/1 and BeninΔMGF, were described previously [6], [9]. Virus titres were shown as the amount of virus infecting 50% of the macrophages cultures (TCID_50_/ml).

**Fig. 1:**
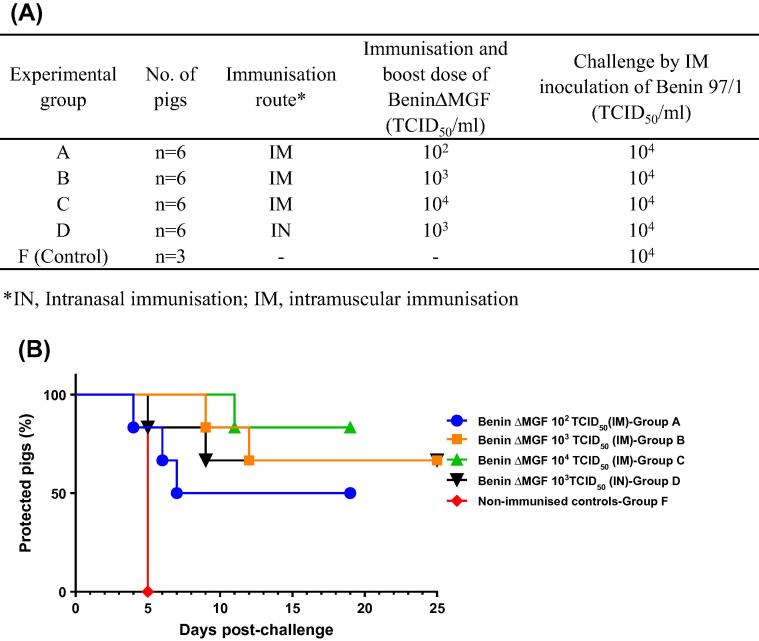
(A) Experimental design. (B) Percentage of protected pigs. Groups of pigs (n = 6) were immunised by intramuscular route (IM) with different doses of deletion mutant BeninΔMFG (10^2^, 10^3^, 10^4^ TCID_50_) and by intranasal route (IN) at 10^3^ dose. Three weeks later all immunised pigs, together with a control group of non-immunised pigs (n = 3), were challenged intramuscularly with 10^4^ TCID_50_ of virulent parental ASFV isolate Benin 91/1. Days post-challenge (x-axis); percentage of protected pigs (y-axis).

Experiments were conducted in SAPO4 high containment facilities at The Pirbright Institute and regulated by the Animals (Scientific Procedures) Act UK 1986. Large White and Landrace crossbred female pigs, 8–9 week-old (18–22 kg), from a high health status herd were used (Fig. 1A). Pigs were separated in groups of six and immunised with BeninΔMGF intramuscularly (IM) in the neck muscles with 1 ml containing 10^2^ (group A), 10^3^ (group B) and 10^4^ (group C) TCID_50_/ml. One group of six pigs (group D) was immunised intranasally (IN) with 2 ml (1 ml per nostril) containing 10^3^ TCID_50_ of BeninΔMGF. At day 21 post-immunisation (pi), pigs were boosted with the same dose and by the same route. After a further 18 days (day 39 pi/day 0 post-challenge, pc), all immunised pigs together with a control group (group F) containing three non-immunised pigs were challenged intramuscularly with 1 ml containing 10^4^ TCID_50_/ml of the parental virulent ASFV isolate Benin 97/1.

### 2.2 Quantitative PCR analysis of virus genome copy numbers

DNA was extracted from whole peripheral blood and analysed for ASFV genome detection by quantitative PCR (qPCR) [8], [10].

### 2.3 Detection of immunoglobulins of isotype M and isotype G and cytokines in swine sera

Serum samples from immunised pigs were analysed using two newly developed ELISA assays (Ingenasa, Madrid; brief protocol described in Fig. 4) based on the semi-purified VP72 protein in order to detect the presence of IgM (capture assay), as an indicator of early infection, and IgG antibodies (indirect assay).

Porcine immunoregulatory cytokines (IFNγ, IL-1β and IL-10; R&D Systems) were assayed in serum samples following manufacturer’s instructions.

## 3 Results

### 3.1 Percentages of protection and clinical signs after immunisation and challenge

The highest percentage of protection (5/6 pig protected; 83%) was achieved in group C (IM, 10^4^ TCID_50_) while group A (IM, 10^2^ TCID_50_) showed the lowest (3/6; 50%) (Fig. 1B).

**Fig. 2:**
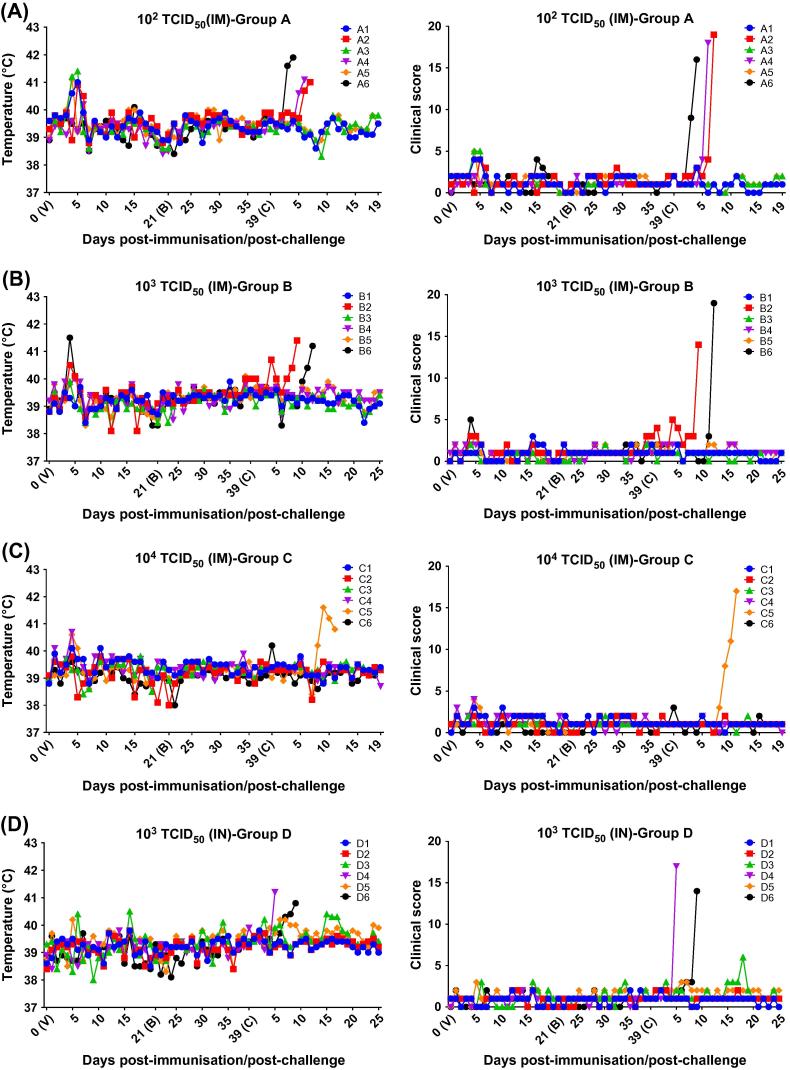
Rectal temperatures and clinical scores in pigs. Rectal temperatures and clinical scores (x-axis) were assessed at different days after immunisation (V), booster (B) and challenge (C) of pigs (y-axis). Pigs were immunised with different doses of deletion mutant BeninΔMFG by intramuscular and intranasal routes: 10^2^ TCID_50_ intramuscular (panel A); 10^3^ TCID_50_ intramuscular (panel B); 10^4^ TCID_50_ intramuscular (panel C); 10^3^ TCID_50_ intranasal (panel D).

Rectal temperatures and clinical signs were monitored as described [8]. A transient increase in temperature was observed in some immunised pigs for 1 or 2 days between days 4 and 6 pi (Fig. 2). In group A (Fig. 2A), 4/6 pigs (A1, A2, A3 and A5) had an increase in rectal temperature above 40.5 °C and in two of these (A1, A3) temperatures increased above 41 °C. In group B (Fig. 2B), only pig B6 displayed a transient increase in temperature post-immunisation (day 4 pi, 41.5 °C). In group C (Fig. 2C), 2 pigs (C4 and C5) displayed a slight transient increase in temperature at day 4 pi (40.7 and 40.6 °C respectively). Finally, in group D (Fig. 2D) none of the pigs showed clinical signs post-immunisation. No further clinical signs or increase in temperature were observed after immunisation or boost in any of pigs.

After challenge, non-immunised control pigs (group F) were euthanized at day 5 pc after reaching a moderate severity end-point. Some immunised pigs in group A (A2, A4 and A6), group B (B2 and B6), group C (C5) and group D (D4 and D6) displayed clinical signs typical of acute ASF and were euthanized between days 4 and 12 pc. No clinical signs were observed in the remaining immunised pigs in any of the groups which were euthanized at day 19 (protected pigs in groups A and C) or 25 (protected pigs in groups B and D) post-challenge.

Statistical analysis showed very significant differences (P < .0001) among mean temperatures of pigs in group A, B and C (10^2^, 10^3^ and 10^4^ TCID_50_; IM) with respect to temperatures of pigs in group D (10^3^ TCID_50_; IN) at days 4 and 5 pi (Supplementary Fig. 1).

### 3.2 Levels of virus genome in blood after immunisation and challenge

**Fig. 3:**
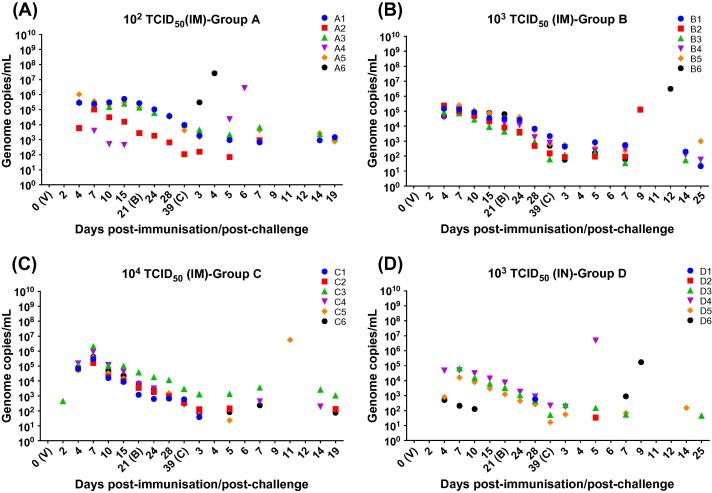
ASFV genome copies in blood samples. EDTA blood samples were collected from all immunised pigs prior and after virus immunisation (V; day 0) and after booster (B). After challenge (C) samples were taken at days 3, 5, 7, 14 as well as at termination of the experiments (at day 19 post-challenge in groups A and C and 25 post-challenge in groups B and D, respectively) (x-axis). Viral genome copies were determined by qPCR and expressed as total genome copies per millilitre (/mL) (y-axis). 10^2^ TCID_50_ intramuscular (panel A); 10^3^ TCID_50_ intramuscular (panel B); 10^4^ TCID_50_ intramuscular (panel C); 10^3^ TCID_50_ intranasal (panel D).

The ASFV genome copy numbers in blood for individual pigs over the course of the experiment are shown (Fig. 3). Pig A6 did not have detectable levels of virus genome before challenge. The rest of the pigs immunised intramuscularly with different doses in group A, B, and C (Fig. 3A-C) had moderate levels of ASFV DNA (10^4^–10^6^ genome copies) in blood by day 4–7 pi, while only 4/6 pigs in group D immunised intranasally (Fig. 3D) had ASFV genome copies (10^2^–10^4^ genome copies) in their blood at this time. Detectable levels of virus genome before challenge were not observed in pig D2, and only detected a very low level at day 28 pi (day 7 after booster immunisation) in pig D1.

A similar trend was recognised in all groups, with genome copy numbers gradually decreasing from day 7 pi until challenge (day 39 pi/0 pc). In group A (Fig. 3A), pig A2 and A4 had lower levels of genome in blood than other pigs in this group (except A6 which had no detectable genome) and virus DNA was not detectable in blood samples of pig A4 from day 15 pi until after challenge. In group D (Fig. 3D), pig D6 also showed lower levels of genome in blood than other pigs except D1 and D2 which had no detectable DNA until after boost or challenge. Genome was not detected in pig D6 from day 15 pi until after challenge.

After challenge (Fig. 3A-D), viremia levels of protected pigs in all immunised groups did not display remarkable changes with respect to pre-challenge values (with values below 10^4^ genome copies) and gradually decreased until the end of the experiment. At termination, viremia levels in protected pigs of group A (A1, A3 and A5) were between 10^2^ and 10^3^ genome copies. In group B only pig B5 displayed values above 10^2^ genome copies. In group C ASFV genome copies were not detected in pigs C1 and C2, and only pig C3 showed values above 10^2^ genome copies. Finally in group D, just one pig (D3) showed detectable levels of genome copies below 10^2^ genome copies/mL at termination.

After challenge, non-protected pigs in all immunised groups showed an increase of virus genome copies that reached levels of 10^5^ to 10^7^ before euthanasia, except pig A2 which has lower levels of genome detected (10^3^). All non-protected pigs were terminated with clinical and pathological signs of acute ASF similar to those described in non-immunised control pigs (group F; 5.45 × 10^7^ to 1.55 × 10^8^ genome copies).

Statistical analysis showed very significant differences (P < .0001) among means of viremia levels of pigs in group A, B and D with respect to viremia levels of pigs in group C (10^4^ TCID_50_; IM) at day 7 pi (Supplementary Fig. 2).

### 3.3 IgM and IgG ASFV specific antibody responses in pigs immunised with BeninΔMGF

**Fig. 4:**
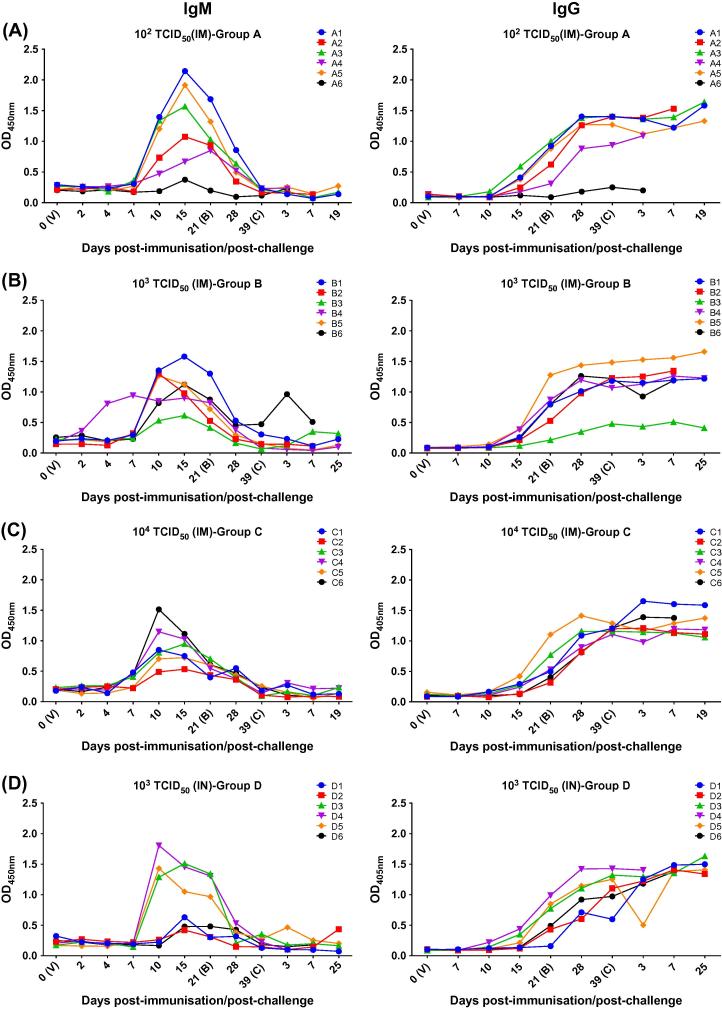
Detection of ASF-specific IgM^1^ (left column) and IgG^2^ (right column) by newly developed ELISA. Serum samples were taken from all immunised pigs prior and after virus immunisation (V; day 0) and after booster (B). After challenge (C) samples were taken at days 3, 7 as well as at termination of the experiments (at day 19 post-challenge in groups A and C and 25 post-challenge in groups B and D, respectively) (x-axis). Optical density (OD) (y-axis). 10^2^ TCID_50_ intramuscular (panel A); 10^3^ TCID_50_ intramuscular (panel B); 10^4^ TCID_50_ intramuscular (panel C); 10^3^ TCID_50_ intranasal (panel D). ^1^(*IgM capture ELISA*): An anti-swine-IgM monoclonal antibody (MAb anti-heavy chain of IgM, produced in INGENASA) was used to coat the plates (1 µg/well) overnight at 4 °C. After blocking for 1 h at RT, the serum samples diluted in PBS-0.5% Tween-20 were added and incubated for 1 h at RT. Subsequently semi-purified VP72 ASFV protein was added (50 ng/well) and incubated for 30 min. at 37 °C. A monoclonal antibody (18BG3, INGENASA) against the VP72 protein and conjugated with peroxidase was then incubated for 30 min. at 37 °C and detected using TMB peroxidase substrate followed by stop buffer (0.5 M sulfuric acid). Enzymatic activity was measured at OD_450nm_ in an ELISA plate reader. Washes between consecutive steps were performed with 0.05% Tween-20 in PBS. ^2^(*IgG indirect ELISA*): The semi-purified VP72 ASFV protein (50 ng/well) was incubated in sodium carbonate buffer (pH 9.6) overnight at 4 °C. After blocking the plates for 1 h at RT, the serum samples diluted in PBS-Tween 0.5% were added and incubated for 1 h at 37 °C. Finally an anti-swine IgG MAb (1BH7, INGENASA) conjugated with peroxidase was incubated for 1 h at RT and detected using ABTS and SDS 0.2% to stop the reaction. The results were read at 405 nm.

IgM seroconversion against VP72 occurred between day 7–10 pi in most animals. Increased levels of IgM remained in serum for 10–18 days and then dropped. IgG antibodies were detected later between day 10–15 pi and remained high for weeks (Fig. 4). In group A, non-protected pigs after challenge (A2, A4 and A6) showed the lowest IgM and IgG levels within the group. IgM and IgG concentrations were especially low in pig A6 which did not display detectable levels of virus genome in blood what suggested this pig might not have become infected. However, non-protected pigs in group B (B2 and B6), group C (C5) and group D (D4 and D6) showed levels of IgM and IgG comparable to those detected in protected pigs. On the contrary, protected pigs D1 and D2 displayed low levels of IgM and IgG comparable to those detected in non-protected pigs.

Statistical analysis did not show significant differences in the mean levels of IgM or IgG among the different immunised groups of pigs or between the group of protected and non-protected pigs (Supplementary Fig. 3).

### 3.4 Levels of IFNγ, IL-10 and IL1-β in sera after immunisation and challenge

**Fig. 5:**
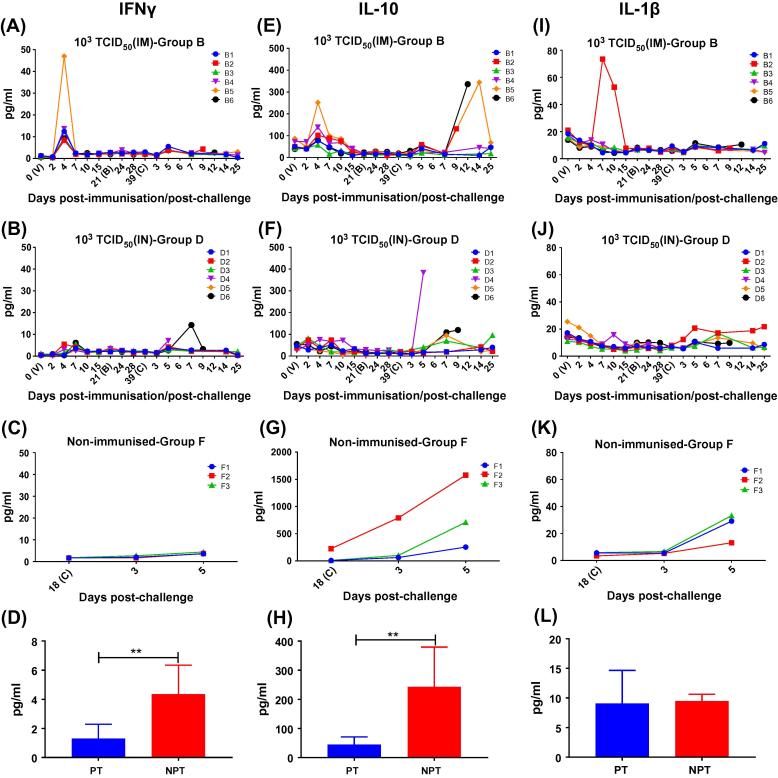
Evaluation of individual serum concentrations of IFNγ (left column; panels A-C), IL-10 (central column; panels E-G) and IL-1β (right column; panels I-K) in samples from pigs immunised with 10^3^ TCID_50_ of BeninΔMFG by intramuscular (group B) or intranasal (group D) route and from non-immunised pigs (group F). Serum samples were collected from all immunised pigs prior and after virus immunisation (V; day 0) and after booster (B). After challenge (C) samples were taken at days 3, 5, 7, 14 as well as at termination of the experiments (at day 19 post-challenge in groups A and C and 25 post-challenge in groups B and D, respectively) (x-axis). Cytokine concentrations were expressed as pg/ml (y-axis). Serum concentrations (mean and SD) of IFNγ (panel D), IL-10 (panel H) and IL-1β (panel L) detected in samples taken just before euthanasia in the group of pigs non-protected (including immunised pigs euthanized between 5 and 12 days pc and non-immunised pigs euthanized at day 5 pc) and in the group of protected pigs euthanized at termination. Statistical analysis was carried out using a Mann-Whitney *U* test for non-parametric distributions. Asterisks indicate statistically significant differences between groups of pigs (^**^P < .01). Protected pigs (PT); Non-protected pigs (NPT).

Serum levels of IFN-γ, IL-10 and IL-1β were evaluated by ELISA in the group B (10^3^ TCID_50_, IM) and D (10^3^ TCID_50_, IN) during the immunisation, boost and challenge and in the control group (Group F) after challenge (Fig. 5). In group B, all pigs showed an increase of IFN-γ levels on day 4 pi (between 46.9 pg/ml in pig B5 and 8.3 pg/ml in pig B2), whereas in pigs of group D a mild increase was observed at day 4 (5.5 pg/ml pig D2; 3 pg/ml pig D4) and 7 pi (3.6 pg/ml pig D1; 5.4 pg/ml pig D3; 2.7 pg/ml pig D5 and 6.1 pg/ml pig D6) (Fig. 5A-B). The mean concentrations of IFN-γ showed a similar trend after primary and booster vaccinations in both groups of immunised pigs. Significantly higher levels of IFN-γ in group B animals compared to those of Group D (P < .0001) were only observed at day 4 pi (Supplementary Fig. 4A). Statistical analysis also showed significant differences in serum concentrations of IFN-γ among samples taken at termination from non-protected pigs and protected pigs (P < .01) (Fig. 5D).

At day 4 pi, an increase of IL-10 levels was observed in pigs of group B. Such increase was higher (above 100 pg/mL) in pigs B2, B4 and B5, levels that were not reached by pigs in group D (Fig. 5 E-F). Statistical analysis confirmed significant differences in mean concentrations of IL-10 between both groups at day 4 pi (P < .0001) (Supplementary Fig. 4B). After day 4 pi, IL-10 mean concentrations in group B decreased and stabilised throughout the primary and booster immunisation time-points without significant differences between the groups. After challenge, significant differences in IL-10 concentrations were not observed between both groups, highlighting the high concentrations reached by pig B5 at day 14 pc that decreased to normal levels before the end of the experiment (25 dpc). Only at termination, IL-10 concentrations were significantly higher (P < .01) in the group of non-protected pigs (Fig. 5H).

Finally, significant changes were not observed in mean concentrations of IL-1β after immunisation or booster (Fig. 5I-J). Individually, only pig B2 displayed higher levels between days 7 and 10 pi. After challenge, significant differences were not detected between both groups or between the group of non-protected and protected pigs at termination (Fig. 5L).

### 3.5 Macroscopic evaluation of lesions and histopathological study

**Fig. 6:**
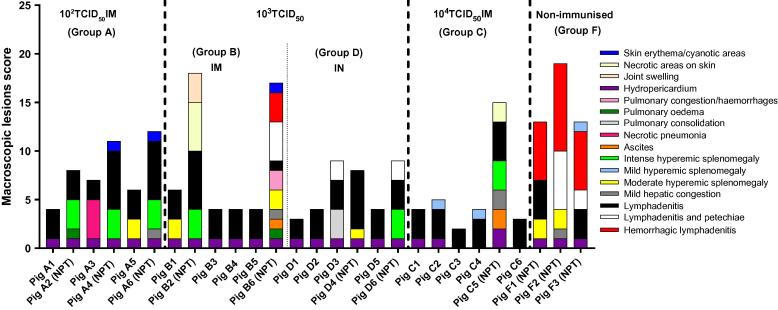
Scoring of macroscopic lesions. The tissues evaluated and the gross lesions observed have been indicated on the graph by different colours. Score of macroscopic lesions (y-axis); pigs evaluated in each of experimental groups (x-axis). NPT: non-protected pigs.

Gross lesions were assessed in all pigs during necropsies as described [11]. Both non-immunised control pigs and non-protected immunised pigs displayed characteristic macroscopic lesions of acute forms of ASF. On the contrary, protected pigs did not display significant gross lesions with the exception of mild hydropericardium and generalized lymphadenitis (Fig. 6). Only some pigs showed other lesions, generally small and localized in specific organs.

Histopathology confirmed a chronic active fibrinonecrotic bronchopneumonia in pig A3 and a diffuse interstitial pneumonia in pig D3, mild lesions compatible with bacterial infection. Histopathologic study also revealed active lymph nodes with the presence of secondary lymphoid follicles and circulating lymphocytes in most of the lymph nodes and tonsils, ruling out any state of immunosuppression that might compromise immune response in protected pigs.

## 4 Discussion

The present study aimed to further evaluate the safety and efficacy of the BeninΔMGF genotype I potential vaccine candidate. Pigs immunised and boosted intramuscularly with 10^4^ TCID_50_ showed the highest percentage of protection (83%). Results were close to described in previous intramuscular immunisations with BeninΔMFG where 100% protection was achieve [6]. However, lower protection was achieved after intramuscular immunisation with 10^2^ TCID_50_ (50%) or with 10^3^ TCID_50_ by both intramuscular and intranasal route (66%). Non-protected pigs immunised intramuscularly with 10^2^ were euthanized earlier between days 4 and 7 pc, while those non-protected pigs immunised by intramuscular route with higher doses (10^3^ and 10^4^) were euthanized later between days 9–12 pc. In addition, non-protected pigs immunised intranasally with 10^3^ TCID_50_ were euthanized earlier than non-protected pigs immunised intramuscularly with the same dose. These results highlighted a strong correlation between the doses of BeninΔMFG administered, the protection achieved and the delay in the appearance of clinical signs and the onset of death in non-protected pigs, also pointing out intramuscular route as the best option for immunisation. However, the chance of higher protection induced by higher doses of BeninΔMFG administered intranasally should not be ruled out, a fact previously demonstrated by intranasal immunisation of naturally attenuated ASFV isolate OURT88/3 [12]. Protection levels provided by high doses of different live attenuated vaccines, equivalent or higher to those used in the present study, have been generally higher than those induced by the administration of lower doses [8], [13], [14], [15], [16]. The BeninΔMGF virus has deletions of three additional genes compared to that tested by O’Donnell et al. [7] using the Georgia isolate. We therefore expect that the deletion we describe will be more attenuated. Although we predict that this deletion will also attenuate the Georgia virus it is possible that the degree of attenuation may differ between the viruses due to other genetic variations between the isolates.

Some pigs immunised intramuscularly showed transient increase in temperature and mild clinical signs after immunisation similar to described previously in pigs immunised with BeninΔMFG [6], changes that were not described in pigs immunised by intranasal route. Statistical analysis also confirmed significantly higher temperatures in the groups of pigs immunised intramuscularly with respect to the group immunised intranasally. On the other hand, the group of pigs in which the highest significant levels of viremia were reached (group C, 10^4^ IM) also displayed the highest percentage of protection. In addition, viremia levels reached by pigs immunised intranasally (group D, 10^3^ TCID_50_) were lower than in groups immunised intramuscularly, including those groups immunised with same or lower doses. These results suggest the existence of differences in ASFV replication mechanisms after immunisation influenced not only by the dose but also by the route of administration, which may influence the appearance of different clinical signs and contribute to trigger different protection mechanisms. In agreement with previous studies where comparative intranasal and intramuscular immunisations against ASFV were carried out [12], the obvious advantage of intranasal delivery was in stimulating the local mucosal immunity although this method of delivery had the disadvantage of being less reliable.

Viremia levels progressively decreased in all groups after day 7 pi. However, virus genome was detected at lower levels for an extended period of time, which in some protected pigs lasted until termination. A similar trend has been recently described in protected pigs vaccinated with a new deletion mutant, Benin DP148R [17] and attributed to the ability of viral particles to bind to the surface of red blood cells mediated by CD2v protein [18], [19], so that an extended persistence of virus on red cells might reflect a loss of infectivity and replication capacity.

One of the new ELISA tests used in the current study enabled early IgM specific antibody detection after immunisation, while the other one showed a high sensitivity to detect IgG specific antibodies. A comparative study demonstrated a good correlation between the newly developed ELISAs and the commercial ELISA (INgezim PPA Compac, Ingenasa) that detects both IgG and IgM antibodies specific for Vp72 (data not shown), displaying that at early stages of infection, only IgM antibodies can be detected. Although the commercial ELISA is highly sensitive, the combined use of both IgM and IgG assays might constitute a valuable tool not only for epidemiological studies of ASF but also for early detection of new outbreaks. So far, variable and controversial results have been obtained about the existence and role played by neutralizing antibodies in protection against ASFV [20]. In the present study, correlation between protection and serum levels of antibodies was not investigated. In previous studies, our group failed to demonstrate the presence of neutralising antibodies in pigs immunised with BeninΔMGF even by the same route and dose used in the present study [6]. Possibly antibodies which have different functional roles in protection may be induced since previous reports described partial protection by passive transfer of serum from immune to naïve pigs [20].

The immunological mechanisms at local or systemic level involved in protection, including the role of cytokines in immune response modulation, have not been elucidated so far. Immunisations of pigs with the same dose of BeninΔMGF (10^3^ TCID_50_) by both intramuscular and intranasal route induced an increase of IFNγ in serum samples of all pigs (protected and non-protected), with significantly higher levels detected at day 4 pi in the group immunised intramuscularly (group B). Such differences might influence the delay in the appearance of clinical signs and the onset of death in non-protected pigs from group B. These results corroborated previous studies *in vivo* where similar changes in IFNγ serum concentrations were also observed in pigs immunised intramuscularly with BeninΔMGF that survived after challenge [6], pointing out the possible role of this cytokine to trigger the immunological mechanisms capable to control ASFV infection after challenge, a role which has been also suggested in *vitro* [8], [17], [21], [22]. Also at day 4 pi, pigs in group B (IM, 10^3^ TCID_50_) showed an increase of IL-10 in serum, considered as a powerful anti-inflammatory mediator with a key role in immune response modulation [23]. In previous experimental vaccinations against ASFV [16], [24], the increase of IL-10 was suggested that might contribute to controlling the first rounds of virus replication and mitigating the harmful consequences of an exacerbated inflammatory response characteristic of acute forms of ASF while avoiding inflammation [25].

However, and due to the fact that both protected and non-protected pigs showed an increase of IFNγ and IL-10 in serum after immunisation, protective mechanisms might involve also other chemical mediators and cellular components. At termination, significantly higher levels of both IFNγ and IL-10 were detected in sera from non-protected pigs. These changes, previously described in non-protected pigs [6], [12], provide evidence of a major dysregulation of protective mechanisms as a consequence of an adverse pathological condition incapable of controlling virus replication.

In conclusion, both doses and routes of immunisation of BeninΔMFG were correlated with the percentage of protection achieved, the onset of clinical signs, the viremia levels reached and the onset of death in non-protected pigs, pointing out intramuscular route with high doses as the best option for immunisation. Correlation between protection and serum levels of IgM and IgG antibodies was not demonstrated. Nevertheless, early induction of IFNγ and IL-10 in vaccinated pigs was probably critical to control initial virus replication and trigger the immunological mechanisms that may favour survival after challenge.

## Conflict of interest

The authors declare they have no conflicts of interest.
