# Supplementary material for: Evaluation of protection induced by immunisation of domestic pigs with deletion mutant African swine fever virus BeninΔMGF by different doses and routes
Source: Vaccine. 2018 Jan 29;36(5):707–15. doi: 10.1016/j.vaccine.2017.12.030 (PMC5783716; doi:10.1016/j.vaccine.2017.12.030)
Supplement: Supplementary data 1 [file mmc1.ppt]

## Slide 1
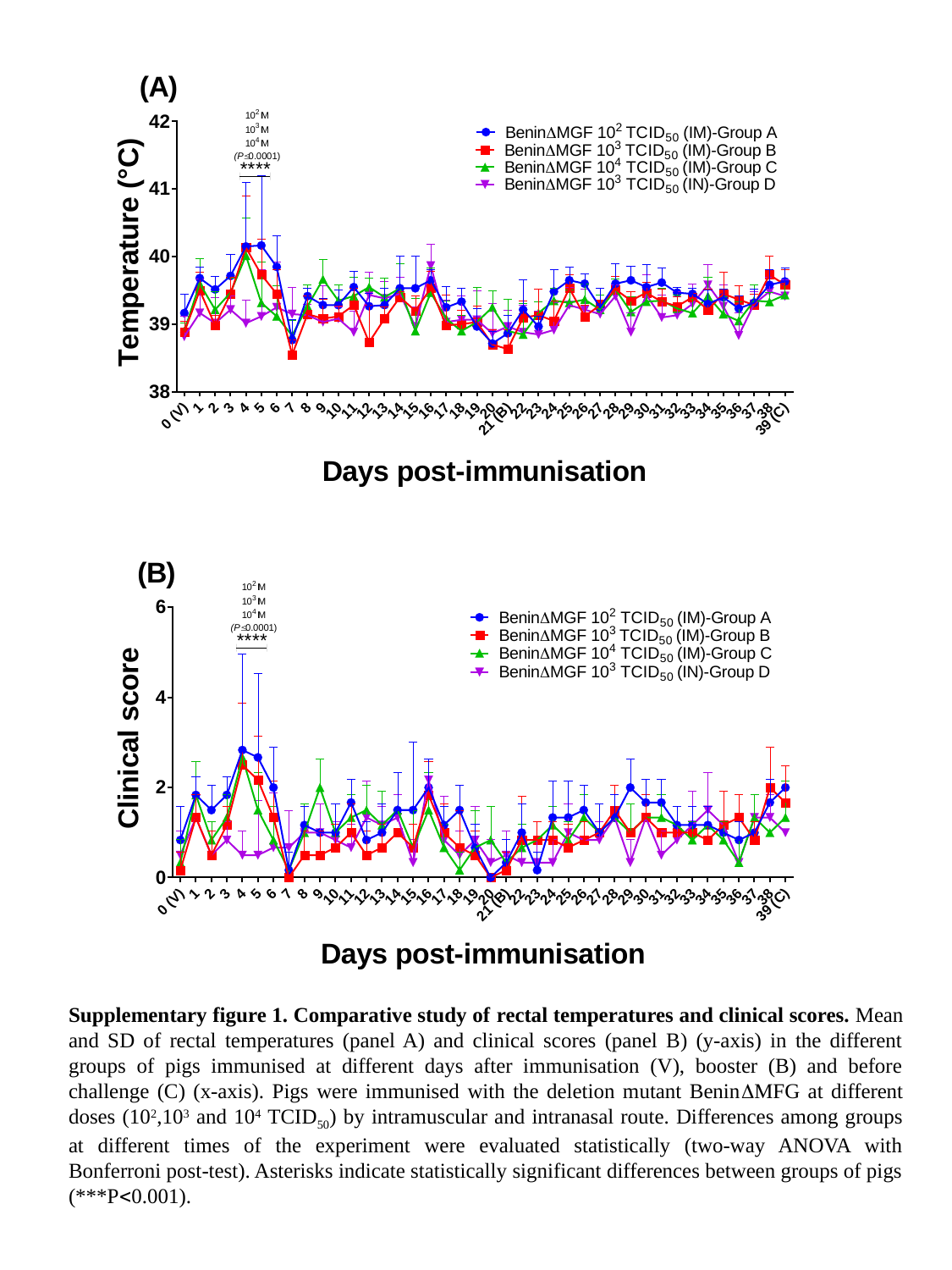

Supplementary figure 1. Comparative study of rectal temperatures and clinical scores. Mean and SD of rectal temperatures (panel A) and clinical scores (panel B) (y-axis) in the different groups of pigs immunised at different days after immunisation (V), booster (B) and before challenge (C) (x-axis). Pigs were immunised with the deletion mutant BeninΔMFG at different doses (102,103 and 104 TCID50) by intramuscular and intranasal route. Differences among groups at different times of the experiment were evaluated statistically (two-way ANOVA with Bonferroni post-test). Asterisks indicate statistically significant differences between groups of pigs (***P0.001).

## Slide 2
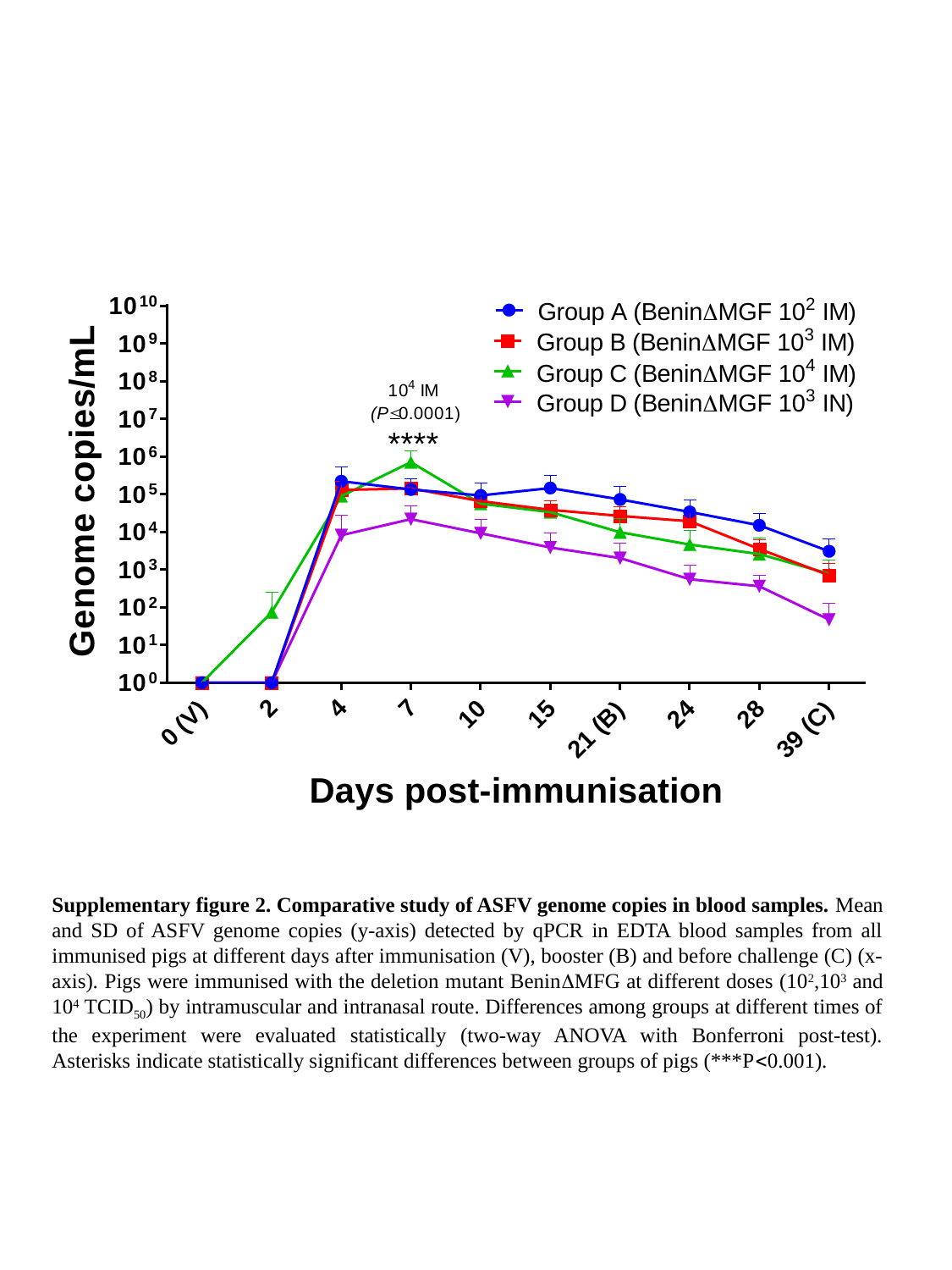

Supplementary figure 2. Comparative study of ASFV genome copies in blood samples. Mean and SD of ASFV genome copies (y-axis) detected by qPCR in EDTA blood samples from all immunised pigs at different days after immunisation (V), booster (B) and before challenge (C) (x-axis). Pigs were immunised with the deletion mutant BeninΔMFG at different doses (102,103 and 104 TCID50) by intramuscular and intranasal route. Differences among groups at different times of the experiment were evaluated statistically (two-way ANOVA with Bonferroni post-test). Asterisks indicate statistically significant differences between groups of pigs (***P0.001).

## Slide 3
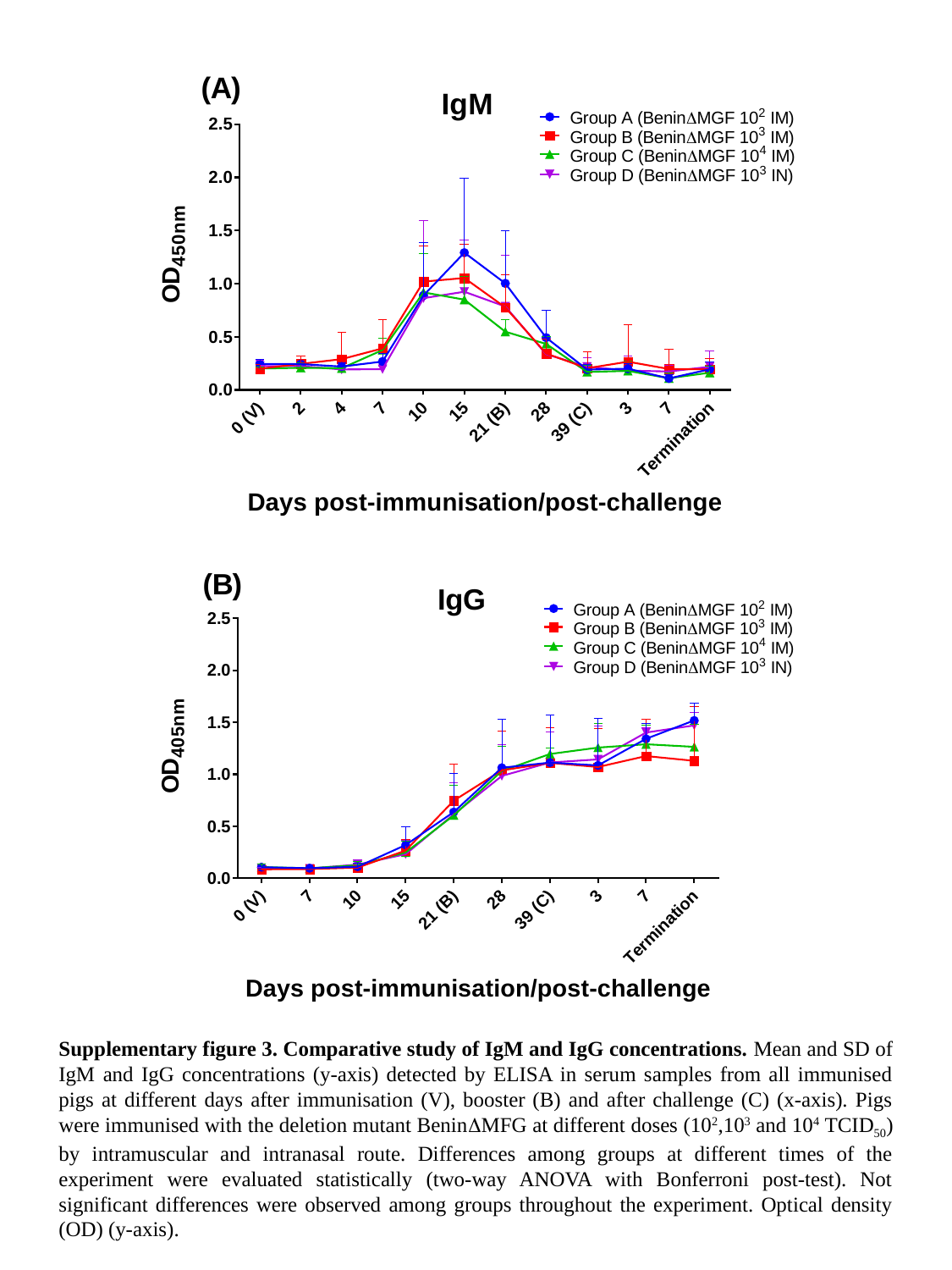

Supplementary figure 3. Comparative study of IgM and IgG concentrations. Mean and SD of IgM and IgG concentrations (y-axis) detected by ELISA in serum samples from all immunised pigs at different days after immunisation (V), booster (B) and after challenge (C) (x-axis). Pigs were immunised with the deletion mutant BeninΔMFG at different doses (102,103 and 104 TCID50) by intramuscular and intranasal route. Differences among groups at different times of the experiment were evaluated statistically (two-way ANOVA with Bonferroni post-test). Not significant differences were observed among groups throughout the experiment. Optical density (OD) (y-axis).

## Slide 4
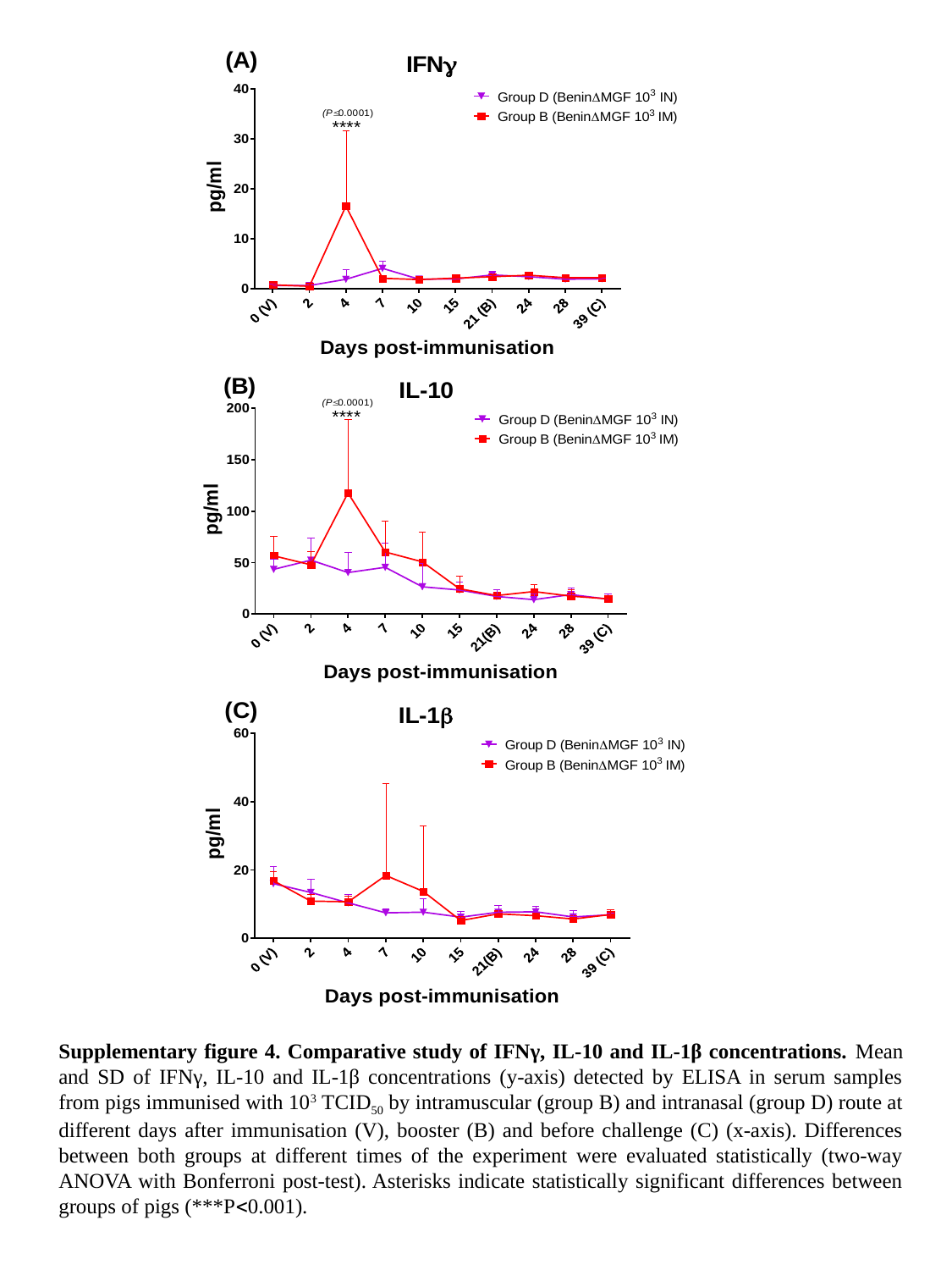

Supplementary figure 4. Comparative study of IFNγ, IL-10 and IL-1β concentrations. Mean and SD of IFNγ, IL-10 and IL-1β concentrations (y-axis) detected by ELISA in serum samples from pigs immunised with 103 TCID50 by intramuscular (group B) and intranasal (group D) route at different days after immunisation (V), booster (B) and before challenge (C) (x-axis). Differences between both groups at different times of the experiment were evaluated statistically (two-way ANOVA with Bonferroni post-test). Asterisks indicate statistically significant differences between groups of pigs (***P0.001).
